# Association between systemic sclerosis and risk of cerebrovascular and cardiovascular disease: a meta-analysis

**DOI:** 10.1038/s41598-024-57275-9

**Published:** 2024-03-18

**Authors:** I-Wen Chen, Wei-Ting Wang, Yi-Chen Lai, Chien-Ming Lin, Ping-Hsin Liu, Su-Zhen Wu, Kuo-Chuan Hung

**Affiliations:** 1 Department of Anesthesiology, Chi Mei Medical Center, Liouying, Tainan, Taiwan; 2 Department of Anesthesiology, E-Da Hospital, I-Shou University, Kaohsiung, Taiwan; 3 Department of Anesthesiology, Chi Mei Medical Center, No. 901, ChungHwa Road, YungKung Dist, Tainan, 71004 Taiwan; 4 Department of Anesthesiology, E-Da Dachang Hospital, I-Shou University, Kaohsiung, Taiwan; 5 Department of Nursing, Min-Hwei Junior College of Health Care Management, Tainan, Taiwan

**Keywords:** Systemic sclerosis, Vasculopathy, Stroke, Cardiovascular disease, Myocardial infarction, Cardiology, Medical research, Neurology, Rheumatology, Risk factors

## Abstract

We aimed to evaluate the association between systemic sclerosis (SSc) and major cerebrovascular/cardiovascular risks through a systematic approach. Databases were systematically searched from their inception to October 10, 2023 for studies comparing cerebrovascular/cardiovascular event rates between patients with SSc and controls. The primary outcome was the stroke risk in patients with SSc. Secondary outcomes included risk of myocardial infarction (MI), cardiovascular disease (CVD), peripheral vascular disease (PVD), and venous thromboembolism (VTE). Seventeen studies with 6,642,297 participants were included. SSc was associated with a significantly increased risk of stroke (HR, 1.64; 95% confidence interval [CI], 1.35–2.01), CVD (HR, 2.12; 95% CI, 1.36–3.3), MI (HR, 2.15; 95% CI, 1.23–3.77), VTE (HR, 2.75; 95% CI, 1.77–4.28), and PVD (HR, 5.23; 95% CI, 4.25–6.45). Subgroup analysis revealed a significantly increased stroke risk in the non-Asian group (HR, 1.55; 95% CI, 1.26–1.9), while the Asian group displayed a higher but not statistically significant risk (HR, 1.86; 95% CI, 0.97–3.55). The study found that SSc is associated with a significantly increased risk of cerebrovascular/cardiovascular events. These findings highlight the importance of vasculopathy in SSc and suggest the need for enhanced clinical monitoring and preventive measures in this high-risk population.

## Introduction

Systemic sclerosis (SSc), also known as scleroderma, is an autoimmune disorder characterized by inflammation, vasculopathy, and excessive collagen deposition^1,2^. Although the thickened, hardened skin is the hallmark of SSc, the disease also frequently affects other organs, including the gastrointestinal tract and lungs^3–5^. A previous meta-analysis has reported a pooled prevalence of SSc of 17.6 per 100,000 and a pooled incidence rate of 1.4 per 100,000 person-years, with noticeable regional disparities; notably, studies from North America reported substantially higher estimates than those of other regions^6^. The disease predominantly affects females, with both pooled incidence and prevalence in females being fivefold higher than those in males^6^. While skin involvement frequently marks the clinical onset of SSc, vascular system dysfunction including vascular injury, vascular remodeling, and endothelial dysfunction represent hallmark pathological changes^7,8^. Systemic vasculopathy can result in various microvascular and macrovascular diseases, thereby leading to multi-organ disorders. Microvascular disease leads to Raynaud’s phenomenon, cutaneous telangiectasia, pulmonary arterial hypertension, scleroderma renal crisis, and gastrointestinal involvement^7–9^. Conversely, macrovascular disease also appears prevalent, with emerging evidence alluding to an increased risk of atherosclerotic events, including stroke, myocardial infarction (MI), and peripheral vascular disease (PVD) in patients with SSc^9–11^.

The risk of cerebrovascular and cardiovascular complications attributed to SSc remains debated^12,13^. Proposed explanations center around accelerated atherosclerosis triggered by chronic inflammation, endothelial dysfunction, and imbalance between vasoconstrictive and vasodilatory mediators^12,13^. Furthermore, SSc vasculopathy could independently precipitate vascular occlusive events^14,15^. Extensive nationwide cohort studies have demonstrated that SSc is independently linked to an increased likelihood of experiencing an ischemic stroke or cardiovascular disease (CVD)^16–18^. Consistently, several meta-analyses have reported the associations of SSc with cerebrovascular and cardiovascular complications^10,19,20^. However, the shortcomings of the earlier evidence stemmed from the inclusion of a restricted number of studies for investigating the associations^10,19,20^. Furthermore, these studies did not investigate the source of heterogeneity^10,19,20^. Recently, several studies have delved into the relationship between SSc and cerebrovascular/CVDs^16,21–24^. These recent studies could provide more solid evidence. In this context, our meta-analysis aimed to synthesize the existing literature on the association between SSc and major cerebrovascular and cardiovascular outcomes. The relationship between SSc and risk of stroke was the primary outcome, whereas the associations of SSc with CVD (e.g., MI), venous thromboembolism (VTE), and other cardiac-related complications (e.g., heart failure) were the secondary outcomes. Elucidating the relationship between SSc and cerebrovascular/CVDs has significant implications for prognosis, screening, prevention, and management. Our findings will help guide clinicians caring for patients with SSc and highlight areas warranting further research.

## Methods

This review was conducted and documented following the MOOSE guidelines, with details of the protocol registration available in PROSPERO (Number: CRD42023471039).

### Search strategy and data sources

An exhaustive and systematic literature search was performed across multiple electronic databases, including Medline, Embase, Cochrane Library, and Google Scholar, spanning from their inception to October 10, 2023. The search strategy was meticulously designed to combine terms specifically associated with SSc (e.g., “scleroderma” and “systemic sclerosis”) with terms indicative of cerebrovascular or CVD (e.g., “coronary artery disease” or “stroke” or “ischemic heart disease” or “myocardial infarction” or “peripheral vascular disease” or “venous thromboembolism”). Adjunctively, to ensure a comprehensive capture of all applicable studies, the reference lists of the articles selected for inclusion, as well as relevant review articles, were manually screened. The search strategy for Medline is summarized in Supplemental Table 1. A similar search strategy was also applied for other databases.

Two independent authors initially screened all identified records from the literature search, first assessing titles and abstracts against predefined criteria. Potentially relevant studies were subsequently subjected to a full-text review. Each author made independent decisions on study inclusion, and any discrepancies were discussed in an attempt to reach consensus. If disagreements persisted, a third expert was consulted. During the selection process, no limitations were placed on the publication year or sample size.

### Inclusion and exclusion criteria

Eligibility for study inclusion was defined as follows: (1) peer-reviewed articles that explicitly reported cerebrovascular/cardiovascular risks (e.g., stroke or MI) among patients diagnosed with SSc and compared these findings to either a control group or the wider general population; and (2) studies that provided hazard ratios (HRs) with 95% confidence intervals (CIs). The following were the exclusion criteria: (1) case reports, case series, review articles, opinion letters, and editorials; (2) any study that lacked a comparative control group or devoid of the necessary quantitative data for analysis; (3) full texts not available; and (4) case–control or cross-sectional studies were not included as they only reported the cumulative risk of cerebrovascular/cardiovascular events (e.g., they provided risk ratios or odds ratios) and not the occurrence of events over time (i.e., HRs).

### Data collection

A pair of independent reviewers, well-versed in the study topic, performed data extraction from the selected studies, employing a standardized and pre-approved data collection template. In instances of disagreements or discrepancies between them, a consensus was sought either through discussion or, if required, mediation by a third senior reviewer. Essential data points extracted encompassed details, including the first author’s name, publication year, total sample size, characteristics of the study population, specific cardiovascular outcomes evaluated (e.g., MI or stroke), and the presented risk estimates with their respective 95% CIs. For studies that provided both unadjusted and adjusted data, we only collected adjusted data for analysis. We gathered the data from the period with the longest follow-up when studies presented identical data across various follow-up periods.

### Outcomes and definitions

The stroke risk in patients with SSc was the primary outcome for this meta-analysis. This outcome was defined as the occurrence of cerebrovascular accidents, including ischemic stroke, transient ischemic attack, or hemorrhage stroke. Moreover, several secondary outcomes were assessed, including the risk of MI, CVD, PVD, VTE, and cardiac-related complications (e.g., heart failure, atrial fibrillation, and pacemaker implantation). The specific definition and criteria for these events were based on the individual definitions provided by each study included in the meta-analysis. As definitions may vary slightly across studies, we took care to ensure consistency in our interpretation and reporting of results.

### Quality assessment of included studies

A rigorous assessment of each study’s methodological quality was performed using the Newcastle–Ottawa Scale (NOS). This assessment critiqued studies across the following three broad domains: the selection process of study groups, the comparability of groups, and the accuracy in ascertaining exposure or outcome data. A maximum score of 9 points denoted the pinnacle of study quality, whereas studies scoring below 6 points were categorized as being of lower methodological quality.

### Statistical analyses

To generate pooled risk estimates (e.g., HRs) with 95% CIs, a random-effects model was employed, anticipating potential heterogeneity across the included studies. A random-effects model was used that weighed studies based on both within-study and between-study variance, not just on sample size. This approach ensures that study weights are not solely proportional to sample sizes, with between-study variance playing a significant role in the weighting process. The degree of heterogeneity was quantified using the I^2^ statistic, with values exceeding 50% signaling significant heterogeneity. To delve deeper into sources of variability, subgroup analysis on the primary outcome (e.g., stroke risk) was performed on the basis of ethnicity (e.g., non-Asian vs. Asian). In addition, to avoid potential patient duplication across studies, a subgroup analysis was performed, including only the single largest cohort study from each country. This subgroup analysis eliminates scenarios in which the same patient can be counted multiple times across different hospitals or insurance datasets within a country. The possibility of publication bias was assessed using funnel plots for outcomes encompassing more than 10 studies. To evaluate the robustness of the results, sensitivity analysis was performed. All statistical analyses were performed using the RevMan software. A p-value of < 0.05 was considered statistically significant.

## Results

### Selection process and characteristics of studies

**Figure 1:**
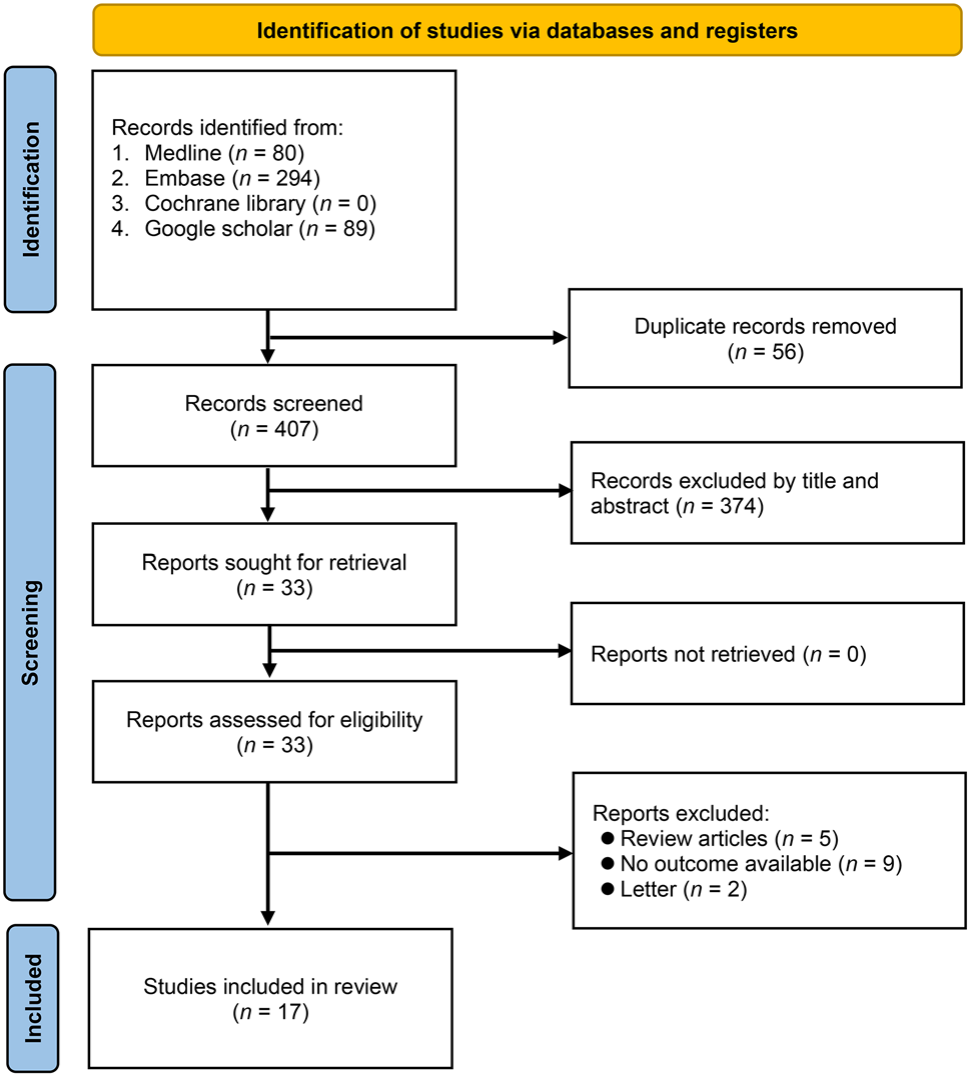
Study selection on the association between systemic sclerosis (SSc) and risk of cerebrovascular and cardiovascular disease (CVD).

From an initial pool of 463 articles identified for the meta-analysis, 56 duplicates were removed, leaving 407 unique articles (Fig. 1). These were preliminarily screened on the basis of titles and abstracts, thereby narrowing the selection to 28 articles for a detailed full-text review. Upon rigorous assessment against specific inclusion and exclusion criteria, 11 articles were excluded for various reasons, resulting in a final set of 17 articles, encompassing a vast participant pool of 6,642,297 individuals, which were deemed suitable and included in the meta-analysis^16–18,21–34^.

The characteristics of studies are summarized in Table 1. We thoroughly examined 17 studies spanning diverse geographic regions, including Canada, Denmark, Taiwan, Belgium, Switzerland, the USA, and Copenhagen. The studies collectively encapsulated a broad participant pool, with the number of patients with SSc ranging from as few as 78 in the study by Kurmann et al.^30^ to as much as 8947 in the study by Thormann et al.^33^. The mean age of participants in these studies varied, with mean ages ranging from 35 years in the study by Thormann et al.^33^. to 60.9 years in the study by Ying et al.^16^. A noteworthy observation across these studies was the prominence of female participants, with percentages frequently exceeding 70%, such as the 91% reported in the study by Kurmann et al.^30^. Additionally, the follow-up duration varied across the studies, with the shortest being 1 year in the study by Thormann et al.^33^, and the longest extending up to 14 years in the study by Aviña-Zubieta et al.^25^.

**Table 1:** Characteristics of studies (*n* = 17) involving 6,642,297 participants.

Study name	Study period	Mean age (years)	Female (%)	*n* (SSc)	*n* (non-SSc)	Outcome	Follow-up (year)	Country	NOS
Aviña-Zubieta 2016	1996–2010	56 vs. 56	83 vs. 83	1239	12,433	a, b, c	14	Canada	9
Butt 2019	1995–2015	55 vs. 55	76 vs. 76	2778	13,520	a, b, c, d, f, g, h	8.9	Denmark	9
Chiang 2013	1997–2006	49.4 vs. 49.4	76 vs 76	1238	12,380	c	4.7	Taiwan	8
Chu 2013	1997–2006	50.6 vs. 50.6	76 vs. 76	1344	13,440	a	4.3 vs. 4.8	Taiwan	8
Chung 2014	1998–2010	50.3 vs. 49.9	75 vs. 75	1895	7580	d	5.3 vs. 6.1	Taiwan	9
Conrad 2022	2000–2017	47.2 vs. 47.6	61 vs. 61	2159	10,310	b	6.2	Belgium	9
Hesselvig 2018	1997–2011	49.2 vs. 40.2	80 vs. 51	1962	5,428,380	b	na	Denmark	5
Huang 2021	2004–2016	52.8 vs. 43.9	72 vs. 50	1507	1,000,000	c	5.1 vs. 5.7	Taiwan	7
Kurmann 2020	1980–2016	56.1 vs. 56	91 vs. 91	78	156	a, b, c, e, f	9.8 vs. 9.2	Switzerland	8
Man 2013	1986–2011	58.7 vs. 58.7	86 vs. 86	865	8643	a, b, c, e,	5.2 vs. 6	USA	8
Michel 2020	2000–2012	na	na	1314	19,992	c	6.5 vs. 7.2	Switzerland	5
Schoenfeld 2016	1996–2010	56.5 vs. 56.5	83 vs. 83	1245	12,670	d	5	USA	7
Sun 2022	1996–2018	55 vs. 55	80.4	1569	6276	a, c, d, f, g, h	7.3	Copenhagen	6
Thormann 2016	1997–2012	35	66 vs. 66	8947	44,735	a, b, c	1	Denmark	9
Tseng 2015	1997–2010	37.1 vs. 37.1	78 vs. 78	1174	4696	c	> 5	Taiwan	7
Yen 2023	1997–2013	53.1 vs. 53.2	71 vs. 71	1379	2758	b	5.2 vs. 6.2	Taiwan	7
Ying 2020	1999–2014	60.9 vs. 61	17 vs. 17	4545	9090	c	5.1 vs. 5.2	USA	7

*na* not available, *SSc* systemic sclerosis, *Outcome* a—myocardial infarction; b—cardiovascular disease; c—stroke; d—venous thromboembolism; e—peripheral vascular disease; f—heart failure; g—atrial fibrillation; h—pacemaker implementation, *NOS* Newcastle–Ottawa Scale.

### Quality of studies

Table 1 summarize the quality of studies by using the NOS. Approximately 53% (9 of 17) of the studies showcased high-quality research methodologies, as indicated by scores ranging from 8 to 9. On the other hand, approximately 41% (7 of 17) of the studies were of moderate quality, with NOS scores between 6 and 7. A small portion, approximately 12% (2 of 17) of the studies, namely Hesselvig 2018 and Michel 2020^29,31^, were identified as having low quality, with scores below 6. This lower score was attributed to potential limitations in participant selection, comparability of groups, or outcome measurement, thereby introducing a greater risk of bias.

### Results of meta-analysis

#### Primary outcome

**Figure 2:**
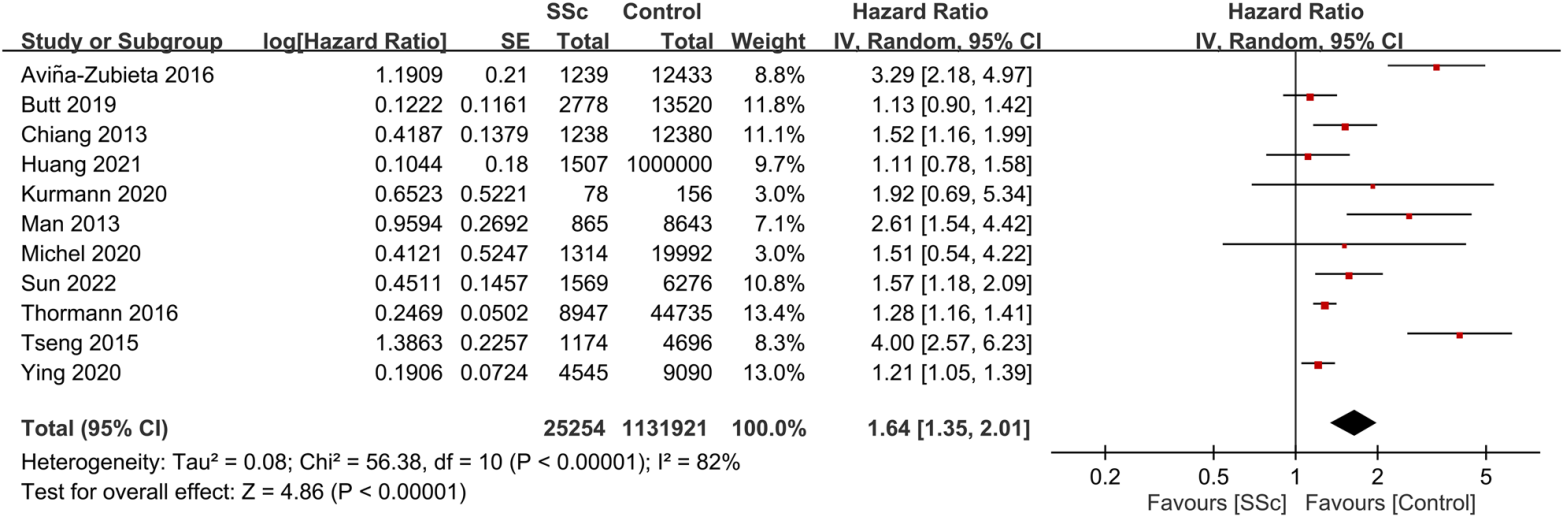
Forest plot showing the association between systemic sclerosis (SSc) and stroke risk. *CI* confidence interval, *IV* inverse variance, *SE* standard error.

**Figure 3:**
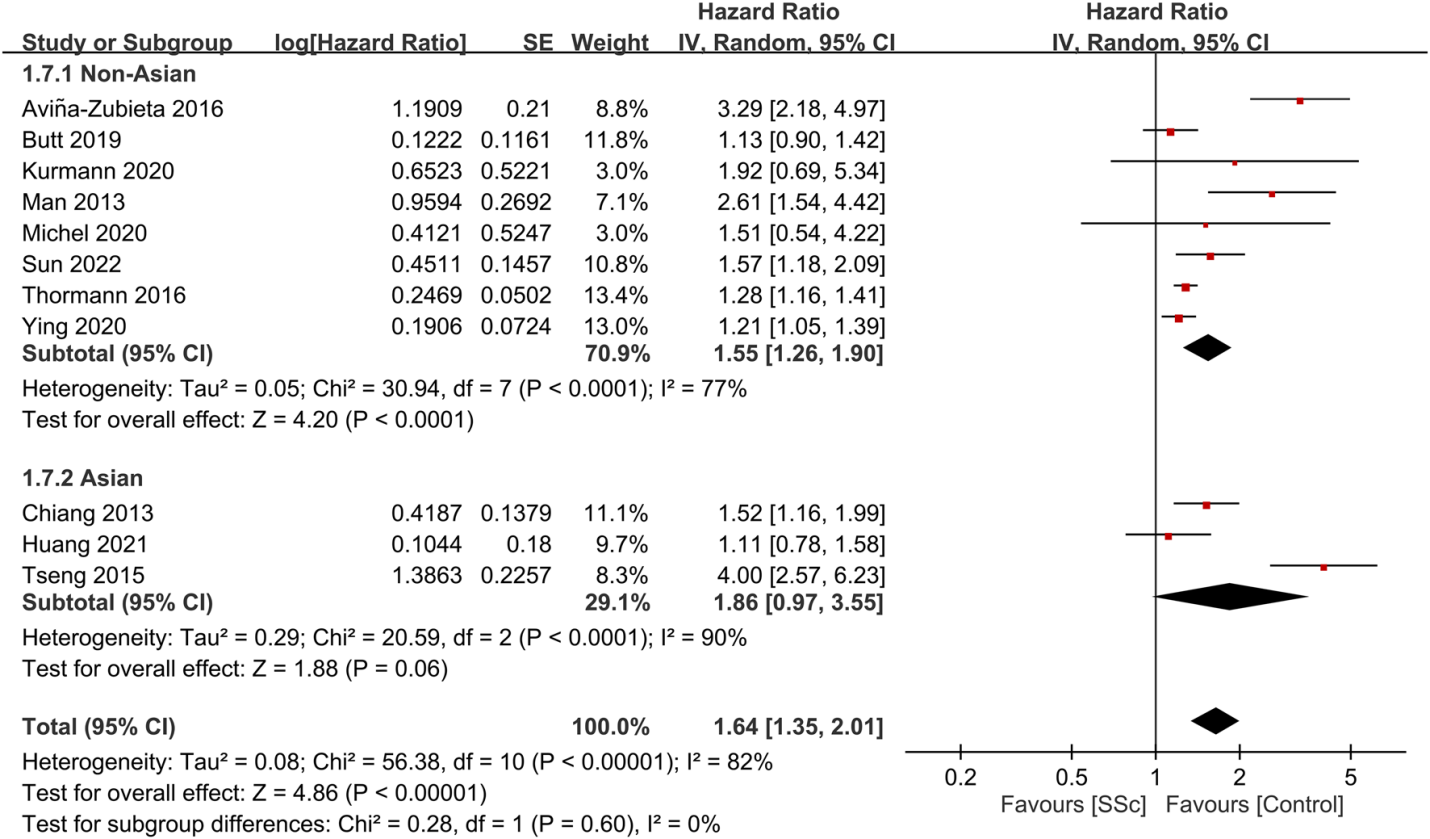
Subgroup analysis on stroke risk based on ethnicity (e.g., non-Asian vs. Asian). *CI* confidence interval, *IV* inverse variance, *SE* standard error.

Among the 17 studies included in our meta-analysis, 11 reported data on the association between SSc and stroke risk^16–18,21,23,25,26,30,31,33,34^. Collectively, these studies encompassed 1,157,175 participants. The pooled HR for stroke in patients with SSc compared with the general population was 1.64 (95% CI, 1.35–2.01; p < 0.00001; I^2^ = 82%) (Fig. 2), suggesting a significant association between SSc and an increased stroke risk. Subgroup analysis indicated a significant increase in risk within the non-Asian subgroup (HR, 1.55; 95% CI, 1.26–1.9; p < 0.0001; I^2^ = 77%). In contrast, while individuals from the Asian subgroup (all from Taiwan)^17,21,24,27,28,34^ demonstrated a trend toward an increased stroke risk, this trend was not statistically significant (HR, 1.86; 95% CI, 0.97–3.55; p = 0.06; I^2^ = 90%) (Fig. 3).

**Figure 4:**
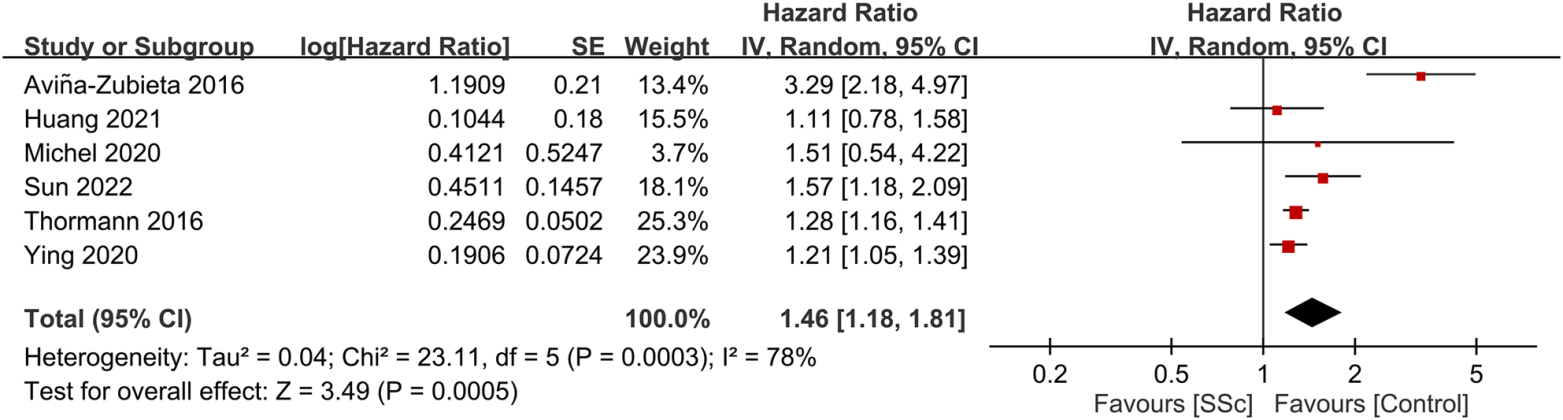
Subgroup analysis of stroke risk in patients with systemic sclerosis (SSc) versus general population using largest cohort studies from each country. *CI* confidence interval, *IV* inverse variance, *SE* standard error.

To evaluate the stability of the outcome, a subgroup analysis was conducted using only the largest cohort study from each country. Therefore, six studies^16,21,23,25,31,33^ with the largest sample sizes from distinct regions were analyzed for the primary outcome. The combined HR for stroke in patients with SSc compared to the general population was found to be 1.46 (95% CI, 1.18–1.81; p = 0.0005; I^2^ = 78%) (Fig. 4), indicating a consistent result.

#### Secondary outcomes

**Figure 5:**
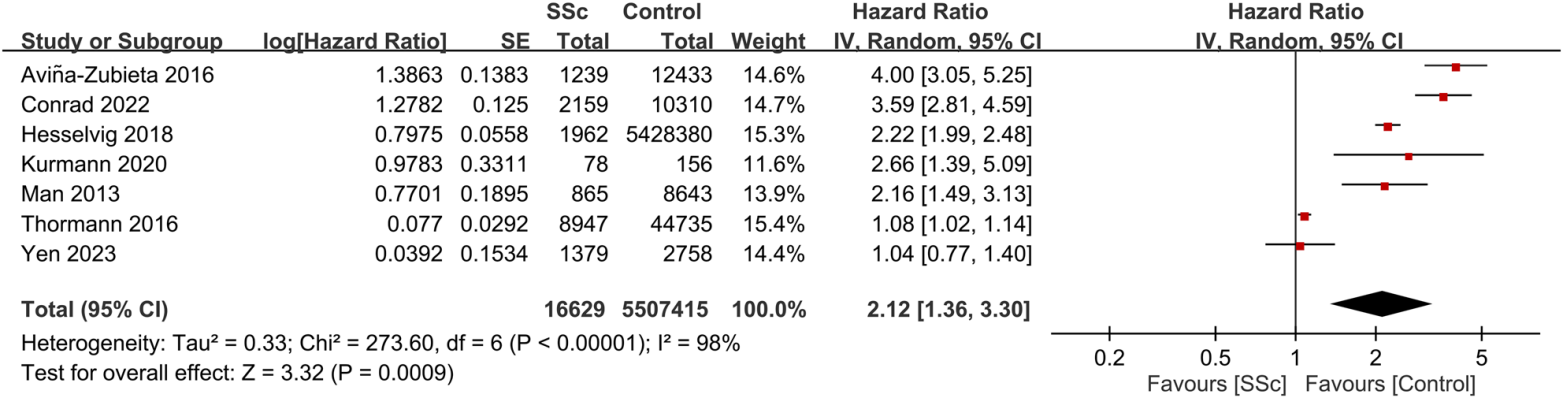
Forest plot showing the association between systemic sclerosis (SSc) and cardiovascular disease (CVD) risk. *CI* confidence interval, *IV* inverse variance, *SE* standard error.

**Figure 6:**
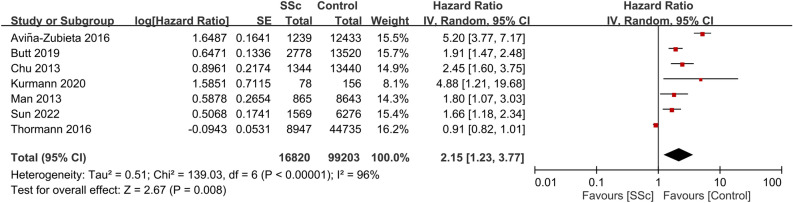
Forest plot showing the association between systemic sclerosis (SSc) and myocardial infarction (MI) risk. *CI* confidence interval, *IV* inverse variance, *SE* standard error.

**Figure 7:**
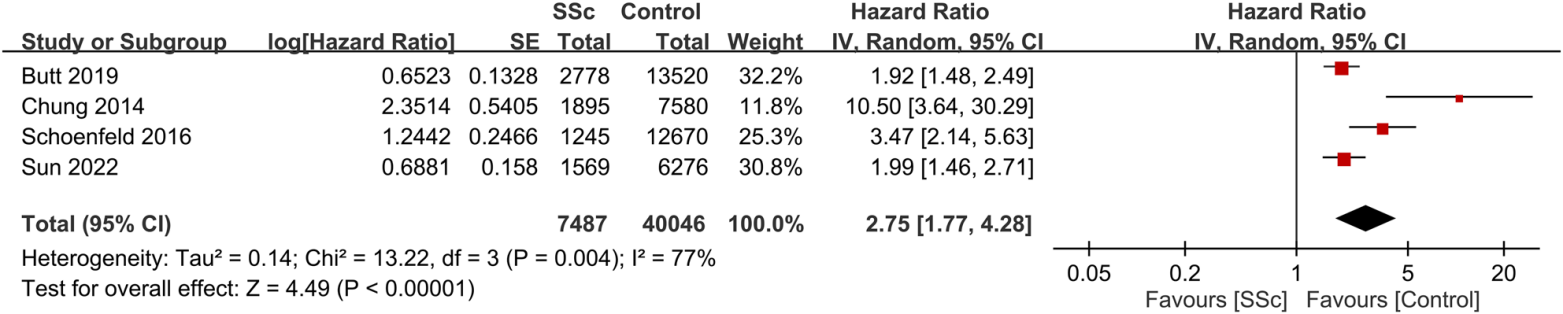
Forest plot showing the association between systemic sclerosis (SSc) and venous thromboembolism (VTE) risk. *CI* confidence interval, *IV* inverse variance, *SE* standard error.

**Figure 8:**
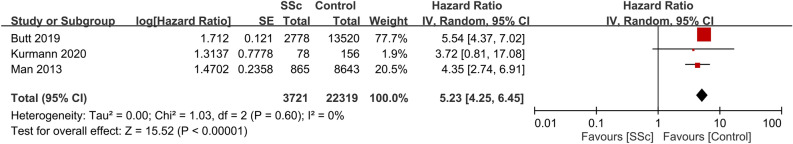
Forest plot showing the association between systemic sclerosis (SSc) and peripheral vascular disease (PVD) risk. *CI* confidence interval, *IV* inverse variance, *SE* standard error.

Seven studies provided data on CVD risk in patients with SSc. Involving a total of 5,524,044 participants, the combined HR for CVD was 2.12 (95% CI, 1.36–3.3; p = 0.0009; I^2^ = 98%)^18,22,24,25,29,30,33^. This suggests that patients with SSc have a significant increased risk of CVD compared with those without the condition (Fig. 5). Consistently, data from seven studies that included 116,023 participants showed a pooled HR of 2.15 (95% CI, 1.23–3.77; p = 0.008; I^2^ = 96%) for MI in patients with SSc, indicating a significant association between SSc and an elevated MI risk (Fig. 6)^18,23,25–27,30,33^. Additionally, patients with SSc were observed to have a heightened VTE risk (HR, 2.75; 95% CI, 1.77–4.28; p < 0.00001; I^2^ = 77%; 47,533 participants) (Fig. 7)^23,26,28,32^. A significantly increased PVD risk was identified among the SSc cohort (HR, 5.23; 95% CI, 4.25–6.45; p < 0.00001; I^2^ = 0%; 26,040 participants) (Fig. 8)^18,26,30^.

**Figure 9:**
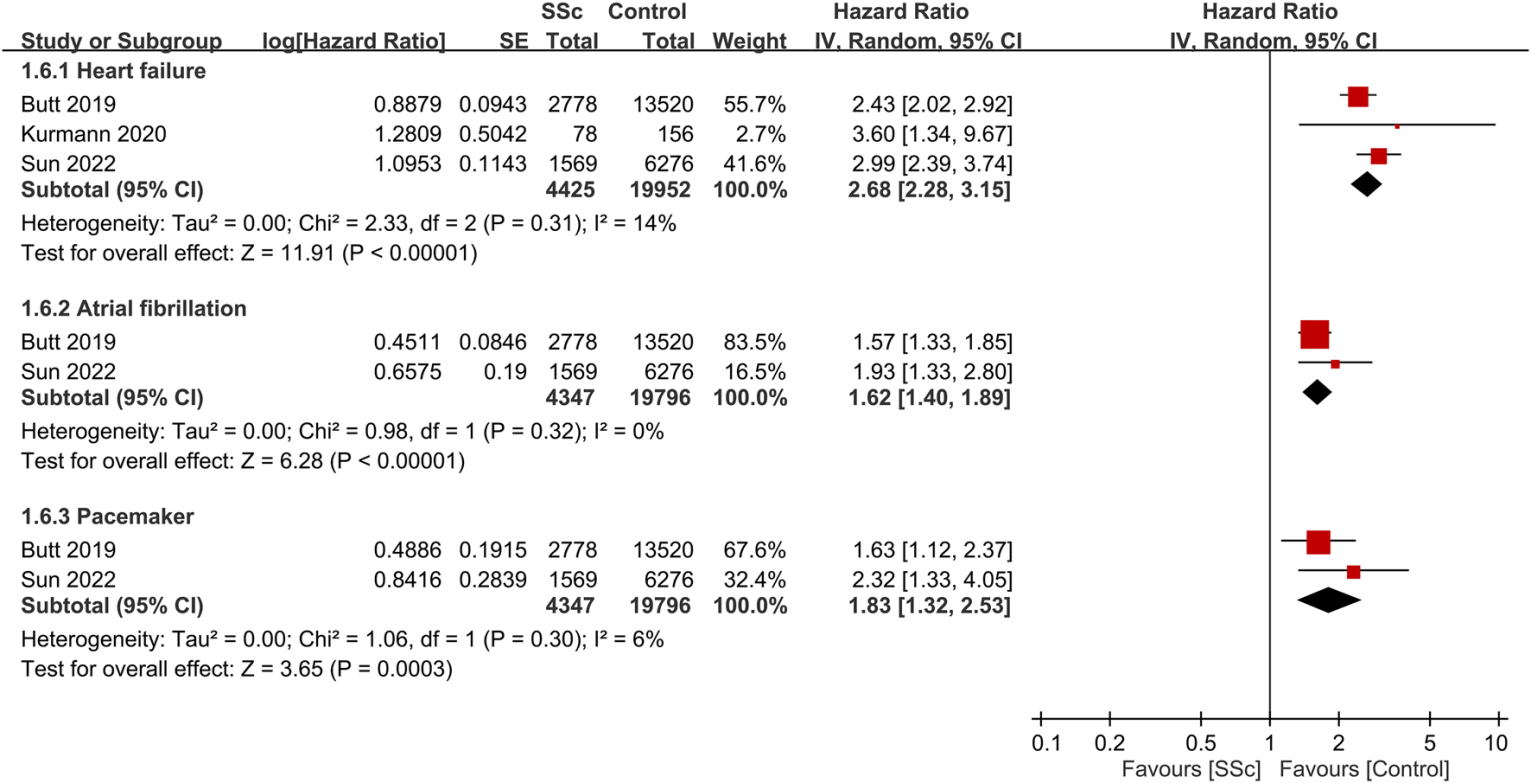
Forest plot showing the association between systemic sclerosis (SSc) and the risks of other cardiac-related complications. *CI* confidence interval, *IV* inverse variance, *SE* standard error.

Regarding other cardiac-related events (Fig. 9)^23,26,30^, heart failure was more prevalent among patients with SSc, with a HR of 2.68 (95% CI: 2.28 to 3.15, p < 0.00001, I^2^ = 14%, 24,377 participants). Atrial fibrillation (AF) occurrence was also higher in the SSc group (HR: 1.62 95% CI: 1.4 to 1.89, p < 0.00001, I^2^ = 0%, 24,143 participants). The risk for pacemaker implantation was elevated among patients with SSc when compared to controls (HR: 1.83 95% CI: 1.32 to 2.53, p = 0.0003, I^2^ = 6%, 24,143 participants).

### Sensitivity analysis

Our sensitivity analysis, aimed at ensuring the robustness of our results, confirmed the consistency of all findings. The omission of any single study from the analysis did not materially alter the overall results. This consistency underscores the reliability of our conclusions and the absence of undue influence by any individual study.

### Funnel plot results

**Figure 10:**
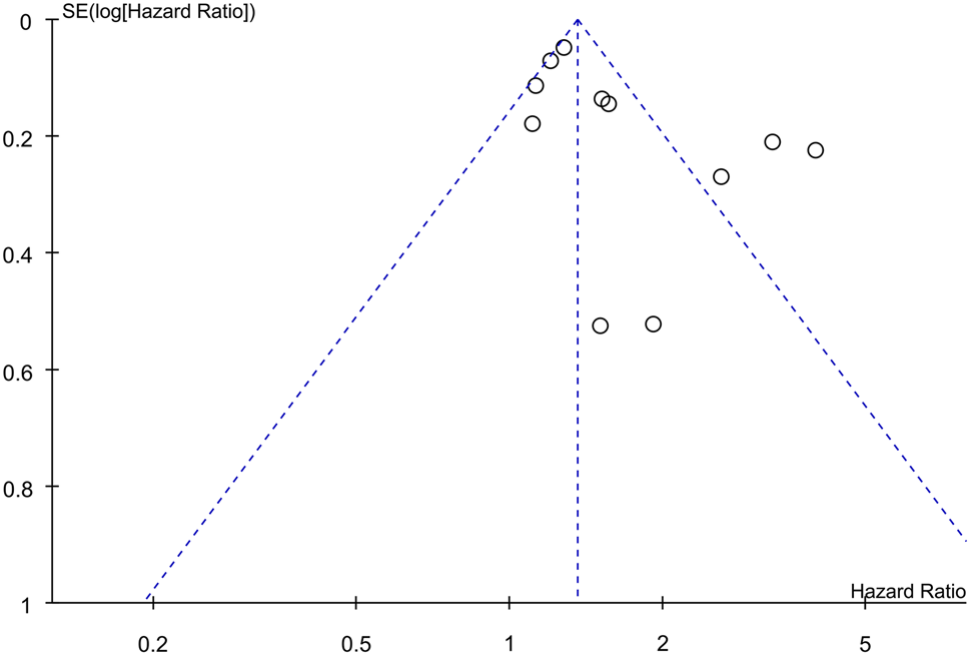
Funnel plots indicating a potential publication bias on the association between systemic sclerosis (SSc) and stroke risk. *CI* confidence interval, *IV* inverse variance, *SE* standard error.

For stroke risk, an evaluation of the funnel plot showcased an asymmetrical dispersion of the included studies, hinting at the presence of significant publication bias (Fig. 10). As there are fewer than 10 studies, funnel plots were not assessed for other outcomes.

## Discussion

Cerebrovascular/CVDs (e.g., MI and stroke) remain dominant contributors to global morbidity and mortality^35^. This systematic review and meta-analysis of 17 studies reported that patients with SSc were at a significantly increased risk of stroke, with a pooled HR of 1.64 (95% CI, 1.35–2.01). Additionally, CVD and MI risks were increased in patients with SSc. Regarding atherosclerotic complications, SSc conferred an added risk of VTE (HR, 2.75) and PVD (HR, 5.23). Patients with SSc had more prevalence of other cardiac manifestations, including heart failure, AF, and pacemaker implantation, than controls. Overall, this meta-analysis demonstrates that SSc is associated with an increased cerebrovascular and CVD risk across a wide spectrum of vascular complications. The elevated risk of both macrovascular events highlights systemic vasculopathy as a central feature of the SSc pathophysiology. Our findings support increased clinical surveillance and consideration of preventative strategies in this high-risk population.

Several meta-analyses have evaluated the association between SSc and stroke risk. Two previous meta-analyses, published in 2016 and 2019, reported a significant association between SSc and stroke risk^10,19^. However, these meta-analyses had limitations as they each reported their main findings on the basis of only four studies. Additionally, these meta-analyses used the cumulative measure of risk (e.g., risk ratio or odds ratio) to examine their association but did not consider the risk of the event over time. A recent meta-analysis assessed stroke risk over time in patients with SSc by including five studies with a total of 67,085 participants^20^. However, only five studies were available for the analysis of the association between SSc and stroke risk in that meta-analysis^20^, which may have impaired the robustness of their findings. In contrast, our meta-analysis included eleven studies with a total of 1,157,175 participants, demonstrating an HR of 1.64. Furthermore, several studies have uncovered racial disparities in SSc presentation^36,37^. Accordingly, another advantage of our meta-analysis lies in the inclusion of a more diverse range of studies, encompassing both Asian and non-Asian populations, which provides more robust evidence.

Our meta-analysis of seven studies with over 116,000 participants noted that patients with SSc had a significantly increased MI risk compared with controls without SSc. The pooled HR was 2.15, indicating that patients with SSc had more than double the risk of experiencing MI. Several mechanisms may explain the observed association between SSc and MI. First, SSc is characterized by chronic inflammation, autoimmunity, and vascular injury^1,2^, which accelerate atherosclerosis and coronary artery disease. Second, endothelial dysfunction and impaired vascular repair, the hallmarks of SSc, also promote atherosclerosis^38,39^. Third, the progression of peripheral microvasculopathy, a key element in SSc pathogenesis, may further contribute to arterial stiffening and myocardial damage over time^40^. Our findings are consistent with a previous meta-analysis demonstrating increased subclinical atherosclerosis, as measured by carotid intima–media thickness, in patients with SSc compared with that in healthy controls^41^. Moreover, clinical studies have reported higher angiographically confirmed coronary artery disease prevalence in asymptomatic SSc^42,43^. The elevated MI risk in SSc remained significant after adjusting for traditional cardiovascular risk factors in several included studies^25–27^, suggesting that disease-related mechanisms mediate much of the increased MI risk.

VTE carries a significant risk of morbidity and mortality, with studies indicating a 30-day mortality rate ranging from 11 to 30%^44–46^. Virchow’s triad is the three principal risk factors for venous thrombosis, including venous stasis, heightened blood coagulability, and vascular wall damage^47^. In patients with SSc, augmented damage to the vascular wall may be observed, as implied by vasculopathy and vascular injury, which are hallmark features of SSc^1,2^. Consistently, our meta-analysis reported a significantly increased risk of VTE among patients with SSc, with a pooled HR of 2.75 compared with individuals without SSc. Our finding is consistent with that of a previous meta-analysis that reported a pooled risk ratio of VTE in patients with SSc being 2.51^48^. The distinction between our study and the previous one^48^ is that we accounted for the potential occurrence of events over time by utilizing the HR. While the mechanisms linking SSc and VTE require further studies, clinicians should be cognizant of the heightened thrombotic risk in this population. Anticoagulation may be considered for VTE prophylaxis in patients with SSc with additional risk factors. Moreover, patients should be educated on promptly reporting VTE symptoms for timely diagnosis and treatment.

Our meta-analysis provides an updated and comprehensive synthesis of the relationship between SSc and major cerebrovascular and cardiovascular outcomes. Involving 17 studies and over 6 million participants, this represents the most extensive meta-analysis on this topic to date. This meta-analysis makes several notable contributions to the literature. First, it provides a contemporary update, incorporating recent large-scale studies published over the past 5 years. Second, it assesses a comprehensive range of clinically relevant cardiovascular outcomes beyond just stroke risk. Third, the expansive dataset of over 6 million participants lends reliability and helps minimize the influence of individual studies. Fourth, subgroup analyses by ethnicity offer initial insights on geographic variations in SSc-related vascular risk. The focus on synthesizing hard clinical endpoints, which represent tangible adverse outcomes in patients, rather than surrogate markers of subclinical vascular dysfunction is the principal novel aspect of this study. Most of the previous literature centered on subtle changes in vascular physiology. Our findings reaffirm vasculopathy as a central SSc feature that warrants greater clinical attention by demonstrating consistent elevations across an array of overt clinical events.

This meta-analysis had several limitations that merit consideration. First, publication bias was a concern, as suggested by the asymmetric funnel plot for the outcome of stroke. It was plausible that small studies demonstrating no significant associations may be underrepresented. Second, inherent to all meta-analyses, the robustness of our conclusions depended on the methodological rigor of the original studies. As all studies were conducted in a retrospective study design, the evidence may be impaired. Third, the reliability of our analyses was restricted by the scarce availability of pertinent clinical details. Data on SSc disease duration, subtype, antibody profiles, severity, and pharmacological treatments were largely unavailable. Without this granular information, elucidating the mechanisms underlying the association between SSc and cerebrovascular/CVD remains challenging. Finally, the statistical power for analyzing some outcomes was limited, as exemplified by the wide confidence intervals or significant heterogeneity. To enable robust conclusions, larger scale prospective studies with adequate adjustment for confounders are needed.

## Conclusion

This meta-analysis, encompassing 17 studies, underscores that patients with SSc face an augmented risk across various vascular complications, including stroke, CVD, MI, VTE, and PVD. Furthermore, these patients exhibited a higher prevalence of other cardiac-related manifestations, including heart failure, AF, and an increased risk of pacemaker implantations. These findings emphasize systemic vasculopathy as a pivotal component of the SSc pathophysiology. Considering these insights, future studies should delve deeper into the mechanistic underpinnings of these associations, explore potential interventions, and assess the efficacy of preventative strategies tailored for this high-risk population.

### Supplementary Information

Supplementary Table 1.

## Data Availability

The original contributions presented in this study are included in this article/Supplementary material, further inquiries can be directed to the corresponding authors.
