# Supplementary material for: Association between systemic sclerosis and risk of cerebrovascular and cardiovascular disease: a meta-analysis
Source: Sci Rep. 2024 Mar 18;14:6445. doi: 10.1038/s41598-024-57275-9 (PMC10948904; doi:10.1038/s41598-024-57275-9)
Supplement: Supplementary file 1 — Supplementary Table 1. [file 41598_2024_57275_MOESM1_ESM.docx]

**Supplemental table 1.** Search strategy

| 1 | ("Systemic sclerosis" or "Scleroderma" or "Systemic Scleroderma").mp. |
| --- | --- |
| 2 | exp "Scleroderma, Systemic"/ |
| 3 | ("Coronary Disease" or "coronary artery disease" or "Coronary Arteriosclerosis" or "Arterial Occlusive Diseases" or "ischemic heart disease" or "Myocardial Infarction" or "Myocardial Infarct" or "Angina" or "Angina Pectoris" or "cardiovascular disease" or "Major Adverse Cardiac Events" or "Stroke" or "Cerebrovascular Accident" or "Venous thromboembolism" or "Thromboembolism" or "Pulmonary Thromboembolism" or "Pulmonary embolism" or "Deep vein thrombosis").mp. |
| 4 | exp "Myocardial Infarction"/ or exp "coronary artery disease"/ or exp "Angina Pectoris"/ or exp "cardiovascular diseases"/ or exp "Stroke"/ or exp "Venous thromboembolism"/ or exp "Pulmonary Embolism"/ or exp "Venous Thrombosis"/ |
| 5 | (1 or 2) and (3 or 4) |
| 6 | (Hazard ratio).mp. |
| 7 | 5 and 6 |
